# Circulating MicroRNAs in Young Patients with Acute Coronary Syndrome

**DOI:** 10.3390/ijms19051467

**Published:** 2018-05-15

**Authors:** Kind-Leng Tong, Ahmad Syadi Mahmood Zuhdi, Wan Azman Wan Ahmad, Paul M. Vanhoutte, Joao Pedro de Magalhaes, Mohd Rais Mustafa, Pooi-Fong Wong

**Affiliations:** 1 Department of Pharmacology, Faculty of Medicine, University of Malaya, 50603 Kuala Lumpur, Malaysia; tkindleng@gmail.com (K.-L.T.); rais@um.edu.my (M.R.M.); 2 Department of Medicine, Faculty of Medicine, University of Malaya, 50603 Kuala Lumpur, Malaysia; syadizuhdi@yahoo.co.uk (A.S.M.Z.); wanazman@ummc.edu.my (W.A.W.A.); 3 State Key Laboratory of Pharmaceutical Biotechnology, Department of Pharmacology and Pharmacy, Li Ka Shing Faculty of Medicine, The University of Hong Kong, Hong Kong, China; vanhoutt@hku.hk; 4 Integrative Genomics of Ageing Group, Institute of Ageing and Chronic Disease, University of Liverpool, Liverpool L7 8TX, UK; jp@senescence.info

**Keywords:** circulating microRNAs, acute coronary syndrome, STEMI, NSTEMI

## Abstract

Circulating microRNAs (miRNAs) hold great potential as novel diagnostic markers for acute coronary syndrome (ACS). This study sought to identify plasma miRNAs that are differentially expressed in young ACS patients (mean age of 38.5 ± 4.3 years) and evaluate their diagnostic potentials. Small RNA sequencing (sRNA-seq) was used to profile plasma miRNAs. Discriminatory power of the miRNAs was determined using receiver operating characteristic (ROC) analysis. Thirteen up-regulated and 16 down-regulated miRNAs were identified in young ACS patients. Quantitative reverse transcription-polymerase chain reaction (qRT-PCR) validation showed miR-183-5p was significantly up-regulated (8-fold) in ACS patients with non-ST-segment elevated myocardial infarction (NSTEMI) whereas miR-134-5p, miR-15a-5p, and let-7i-5p were significantly down-regulated (5-fold, 7-fold and 3.5-fold, respectively) in patients with ST-segment elevated myocardial infarction (STEMI), compared to the healthy controls. MiR-183-5p had a high discriminatory power to differentiate NSTEMI patients from healthy controls (area under the curve (AUC) of ROC = 0.917). The discriminatory power for STEMI patients was highest with let-7i-5p (AUC = 0.833) followed by miR-134-5p and miR-15a-5p and this further improved (AUC = 0.935) with the three miRNAs combination. Plasma miR-183-5p, miR-134-5p, miR-15a-5p and let-7i-5p are deregulated in STEMI and NSTEMI and could be potentially used to discriminate the two ACS forms.

## 1. Introduction

Coronary artery disease (CAD) is a leading cause of death worldwide. Clinical presentations of CAD include silent ischemia, stable angina pectoris, unstable angina, myocardial infarction, heart failure, and sudden death [1]. Acute coronary syndrome (ACS) refers to any group of clinical symptoms compatible with acute myocardial ischemia and includes unstable angina pectoris, ST-segment elevated myocardial infarction (STEMI) and non-ST-segment elevated myocardial infarction (NSTEMI) [2]. Between the years 2011 and 2013, approximately 15,000 ACS cases were reported in Malaysia, with more than 75% afflicting patients older than 50 years of age. To date, the rates of myocardial infarction, readmission of patients with ACS and death remain high, underscoring the need for more aggressive health awareness, education, and non-pharmacological approaches to counter this rising prevalence of ACS as well as innovative early and reliable biomarkers to facilitate rapid and more accurate diagnosis and subsequent treatment.

Electrocardiography (ECG) and cardiac troponins are conventional tests used for the diagnosis of ACS. ECG detects transient ST-segment elevations, dynamic T-wave changes, and ST depressions during acute events [3]; however, ECG patterns that are diagnostic of ACS are not present in approximately half of the ACS patients at the time of presentation in an emergency department [4]. Hence a significant proportion of ACS may elude detection by ECG alone. For this reason, the diagnosis of ACS remains a challenge especially in individuals without clear symptoms or electrocardiographic features.

Cardiac troponin I or T are not detectable in the blood of healthy individuals and are more predictive of myocardial necrosis compared to creatine kinase-myocardial band isoenzyme (CK-MB) [5]. However, cardiac troponins may not be elevated consistently at the onset of myocardial infarction, which can hinder early diagnosis [6]. Moreover, changes in troponin levels also occur under conditions other than ACS (including chronic renal failure, cerebrovascular accidents, acute pulmonary embolism, hypertensive crisis, tachy- or bradyarrhythmias, acute inflammatory myocarditis, and skeletal myopathies) [7]. Misinterpretation of detectable troponin levels may lead to confusion of diagnosis and inappropriate choice of therapy. Hence, there is a need to identify additional biomarkers to support accurate clinical diagnosis and management.

MicroRNAs (miRNAs) have been identified and advocated as biomarkers for the diagnosis and/or prognosis of cardiovascular diseases such as ischemic heart disease, heart failure, stroke, and CAD [8,9]. They are short (≈22 nucleotides), non-coding RNAs that regulate genes post-transcriptionally by translational repression following partial complementary binding to target sites on untranslated regions of mRNAs [10,11,12]. MiRNAs play important roles in various biological and cellular processes by regulating the expression of their target genes in signaling pathways. They are present in tissues and in the circulation; aberrations of miRNAs expression levels occur in various diseases including cancer and cardiovascular disorders [13,14,15,16]. In the latter, for example, the expressions of miR-134, miR-198, and miR-370 are increased in patients with unstable angina pectoris and acute pulmonary embolism [17,18]. The use of circulating miRNAs for the diagnosis of diseases is reliable as they are stable in plasma or serum and their expressions can be easily quantified by quantitative reverse transcription-polymerase chain reaction (qRT-PCR). In this regard, it would be advantageous to detect early alterations in the levels of circulating miRNAs for the diagnosis of ACS during the early onset of symptoms. This is exemplified by the detection of circulating miRNAs within three hours of symptom onset and in patients with initially negative troponin levels [19]. Specifically, peak expression levels of miR-19b-5p, miR-134-5p, and miR-186-5p can be detected approximately ten hours after the onset of chest pain in patients with acute myocardial infarction [20]. Moreover, their presence also positively correlates with cardiac troponin I. Given that a single miRNA can have various targets and in view of the cooperative mechanisms involved, a multi panel approach of miRNAs detection could further increase diagnostic efficiency. Taken in conjunction, the available information suggests that circulating levels of certain miRNAs have strong potentials as biomarkers for the diagnosis of cardiovascular diseases.

In the diagnosis of ACS, the identification and evaluation of the use of circulating miRNAs have thus far focused on patients of higher age groups, as older people are more at risk for ACS where its prevalence is indeed higher in the elderly [20,21,22]. Apart from age, other risk factors for ACS include smoking, diabetes, hypertension, obesity, and hypercholesterolemia [23,24]. In Malaysia, approximately 23% of ACS cases involved individuals below 50 years of age and the majority of these patients were male (~80%) [25]. In addition, a retrospective review in the northern region of Malaysia further revealed that 55% of these patients had premature CAD [26]. The increasing prevalence of ACS in younger individuals could be linked to the continuous rise in obesity, diabetes, and hypertension in that age group in Malaysia. The occurrence of ACS in young individuals is uncommon and is often overlooked during clinical examination but the prognosis is more favorable compared to that of elderly patients [27,28]. Hence, there is a need to identify suitable early biomarkers for young ACS patients as they are often under-diagnosed due to a lower clinical suspicion. The present study sought to achieve this by performing small RNA sequencing to identify plasma miRNAs in young ACS patients.

## 2. Results

### 2.1. Baseline Characteristics of the Study Population

Assessment of clinical characteristics revealed no statistical difference between the healthy control subjects with the ACS subgroups for most of the clinical variables measured except for significantly higher plasma levels of high-density lipoproteins (HDL) and lower white blood cell counts (WBC) in the healthy controls compared to the ACS group (Table 1 and Table 2). This is anticipated as lower plasma HDL level in patients is associated with the incidence of premature CAD [26,29]. There were also no significant differences in the age (*p* = 0.16) and CK-MB levels (*p* = 0.69) between the ACS and healthy subjects. As expected, patients with STEMI had higher troponin I and CK-MB levels compared to NSTEMI subjects because the degree of coronary occlusion is worse in STEMI (Table 1 and Table 2). The percentage of patients prescribed with medications was also higher in the STEMI than in the NSTEMI group. This reflects that ACS with STEMI, which has a worse outcome, warrants more aggressive treatment.

### 2.2. Detection of miRNA Expression in Plasma of Young ACS by Small RNA Sequencing (sRNA-seq)

**Figure 1:**
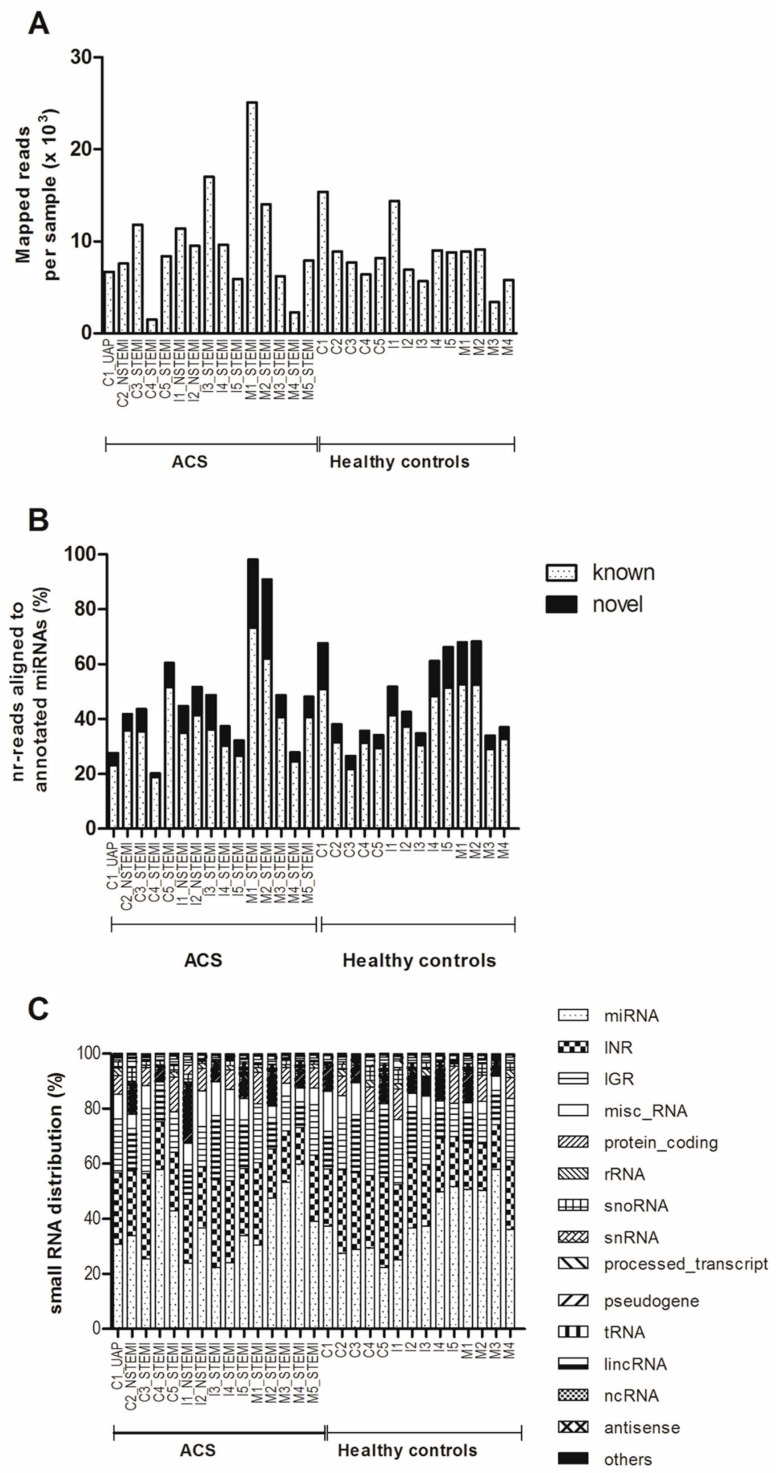
Overview of mapped reads, distribution of RNA species and miRNAs. (**A**) Number of reads (×10^3^) mapped to the human genome for all samples. These include all RNA species [small nucleolar (sno), small nuclear (sn), rRNA, tRNA, non-coding (nc), long coding (linc), intergenic (IGR), intronic (INR) and miscellaneous other (misc) RNA]. (**B**) Distribution of the mapped reads in the reference genome (GRCh37). (**C**) Percentages of mapped reads aligned to annotated miRNA genes in the reference genome for all samples.

On the average, 58,000 reads mapped to the human genome were obtained per sample and after applying the filter, an average of 9086 clean reads were obtained (Figure 1A). Length distribution analysis showed that most reads were in the range of 18 to 24 nt in plasma pools of both the ACS patients and the healthy controls, which is consistent with the common length of miRNAs [30,31]. MiRNAs were the major component of the small RNAs population in the dataset (Figure 1B). MiRDeep2 analysis identified a total of 1702 miRNAs, of which 752 are known, from the pooled samples. On the average, 38.5% of mapped nr-reads were aligned to annotated miRNA genes in the reference genome (Figure 1C). In the STEMI group, a total of 166 miRNAs with reads above 200 and present in more than five subjects were retained in the list used for differential expression analysis using DESeq2 (https://bioconductor.org/packages/release/bioc/html/DESeq2.html) [32]. A cut-off of 10% false discovery rate (FDR) yielded 13 up-regulated and 16 down-regulated miRNAs in the STEMI group (Table 3). MiRNAs with more than 1.5-fold changes in expression (i.e., miR-183-5p, miR-15a-5p, miR-375, miR-1307-5p, miR-134-5p, and let-7i-5p) were selected for subsequent qRT-PCR validation.

### 2.3. Quantitative RT-PCR (qRT-PCR) Validation of miRNAs Expression

**Figure 2:**
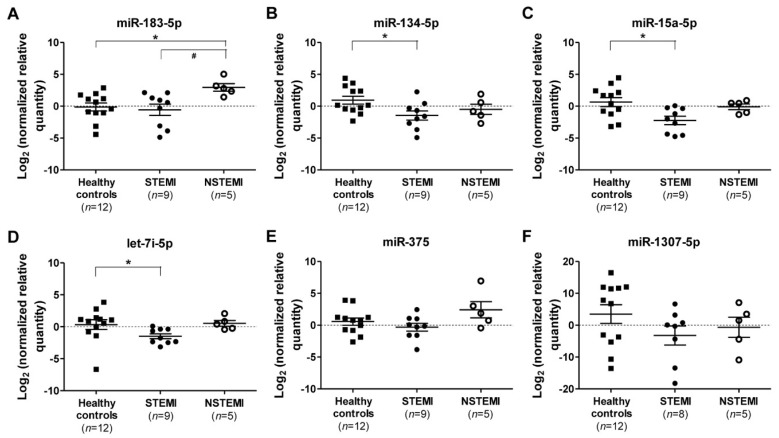
MiRNAs expression in ACS subtypes. Log_2_ transformation of normalized relative quantity of (**A**) miR-183-5p, (**B**) miR-134-5p, (**C**) miR-15a-5p, (**D**) let-7i-5p, (**E**) miR-375, and (**F**) miR-1307-5p in healthy controls (*n* = 12), STEMI (*n* = 9), and NSTEMI (*n* = 5) patients. The expressions of the target miRNAs were normalized to both reference miRNAs, i.e., miR-320a and miR-17-5p. Data are shown as the geometric means ± 95% CI, confidence interval. *p*-values were calculated using one-way ANOVA followed by the Tukey multiple groups comparison test; * indicates *p* < 0.05 compared to healthy control individuals; and ^#^ indicates *p* < 0.05 compared to NSTEMI patients.

A second cohort of young male patients was recruited for qRT-PCR validation (Table 2), including two STEMI, two NSTEMI patients, and nine healthy controls from the original sRNA-seq cohort. Validation by qRT-PCR revealed a significant 8-fold up-regulation of miR-183-5p in NSTEMI patients (*p* = 0.024) compared to healthy controls (Figure 2A). The expressions of miR-183-5p in NSTEMI patients were also significantly higher than those of the STEMI patients (Figure 2A). In STEMI patients, miR-134-5p, miR-15a-5p, and let-7i-5p were significantly down-regulated 5-fold (*p* = 0.048), 7-fold (*p* = 0.015) and 3.5-fold (*p* = 0.013), respectively, compared to healthy controls (Figure 2B–D), whereas miR-375 and miR-1307-5p were not significantly differentially expressed in STEMI and NSTEMI patients compared to the healthy controls (Figure 2E,F).

**Figure 3:**
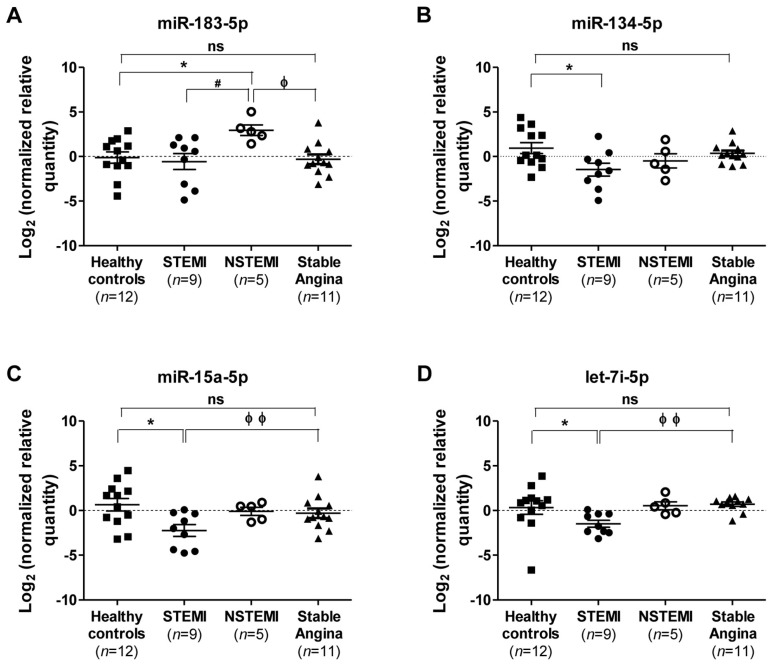
Comparison of miRNAs expression in individuals with ACS and stable angina. Log_2_ transformation of normalized relative quantity of (**A**) miR-183-5p, (**B**) miR-134-5p, (**C**) miR-15a-5p, and (**D**) let-7i-5p in healthy controls (*n* = 12), STEMI (*n* = 9), NSTEMI (*n* = 5), and stable angina (*n* = 11) patients. The expressions of the target miRNAs were normalized to both reference miRNAs, i.e., miR-320a and miR-17-5p. Data are shown as the geometric means ± 95% CI, confidence interval. *p*-values were calculated using one-way ANOVA followed by the Tukey multiple groups comparison test; ns indicates not significant; * indicates *p* < 0.05 compared to healthy control individuals; ^#^ indicates *p* < 0.05 compared to NSTEMI patients; ϕ indicates *p* < 0.05 and ϕϕ, *p* < 0.01 compared to stable angina patients.

Since miR-183-5p, miR-134-5p, miR-15a-5p, and let-7i-5p were deregulated significantly in STEMI and NSTEMI patients, their expressions were compared further with those of the stable angina patients without ACS for more than one year. The expressions of miR-183-5p, miR-134-5p, miR-15a-5p, and let-7i-5p were not significantly deregulated in the stable angina patients compared to healthy controls (Figure 3). One-way analysis of variance (ANOVA) analysis followed by the Tukey multiple comparison test revealed that miR-183-5p is significantly up-regulated in NSTEMI compared to the stable angina patients (Figure 3A). In addition, the expression of miR-15a-5p and let-7i-5p were significantly down-regulated in patients with STEMI compared to those with stable angina (Figure 3C,D). The expression of miR-134-5p for STEMI patients was only significantly down-regulated when compared to healthy controls but not to the NSTEMI and stable angina groups (Figure 3B).

### 2.4. Diagnostic Power of Plasma miRNAs in STEMI and NSTEMI Patients

**Figure 4:**
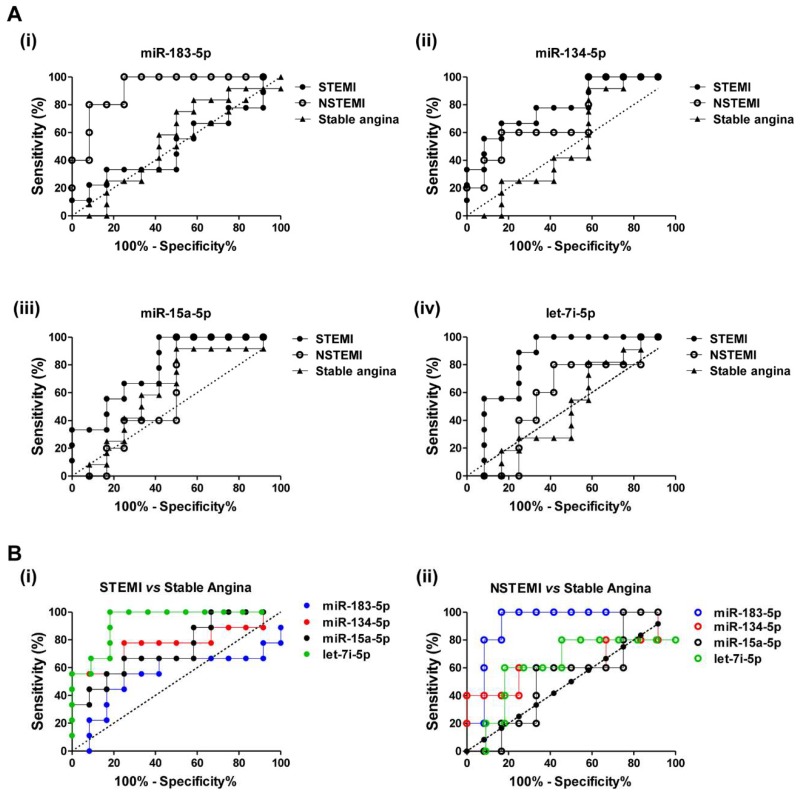
Discriminatory power of plasma miRNAs for ACS patients. (**A**) Receiver operator characteristic (ROC) curves for (i) miR-183-5p, (ii) miR-134-5p, (iii) miR-15a-5p and (iv) let-7i-5p in STEMI, NSTEMI and stable angina patients against healthy controls. (**B**) ROC curves for miR-183-5p, miR-134-5p, miR-15a-5p, and let-7i-5p in (i) STEMI and (ii) NSTEMI patients against patients with stable angina. ROC analysis was performed using GraphPad Prism version 5.0 (GraphPad Software, San Diego, CA, USA).

The diagnostic accuracy and discriminatory power of miR-183-5p, miR-134-5p, miR-15a-5p, and let-7i-5p for patients with STEMI and NSTEMI from healthy controls was determined using receiver operator characteristic (ROC) analysis, and illustrated by area under the curve (AUC) of ROC (Figure 4A and Table 4). MiR-183-5p scored a very high AUC value of 0.917 (*p* < 0.0001; 95% CI, confidence interval 0.680 to 0.995) with sensitivity and specificity values of 100% and 75%, respectively; this indicates a strong differentiation power of miR-183-5p for NSTEMI patients from healthy controls. However, miR-183-5p showed weaker discriminatory power for STEMI patients with an AUC value of 0.509 (*p* = 0.947; 95% CI, 0.285 to 0.731) from the healthy controls (Figure 4A(i) and Table 4). Nevertheless, miR-134-5p, miR-15a-5p, and let-7i-5p showed better differentiation power in discriminating STEMI patients from healthy controls with higher AUC values of 0.796 (*p* = 0.004; 95% CI, 0.566 to 0.938), 0.796 (*p* = 0.003; 95% CI, 0.566 to 0.938), and 0.833 (*p* = 0.0005; 95% CI, 0.608 to 0.958), respectively (Figure 4A(ii)–(iv), Table 4). Moreover, among these three miRNAs, the discriminatory power of let-7i-5p was the highest with a sensitivity value of 100% and a specificity value of 67% in differentiating STEMI patients from healthy controls (Table 4). Collectively, these findings reveal the strong discriminatory power of miR-183-5p in NSTEMI patients and miR-134-5p, miR-15a-5p, and let-7i-5p in STEMI patients from the healthy controls.

To investigate the discriminatory power of circulating miRNAs of ACS patients from patients with stable angina, AUC values for their respective ROC curves were obtained (Figure 4B and Table 5). The discriminatory power of plasma miRNAs in STEMI against stable angina patients was highest in let-7i-5p, followed by miR-134-5p, miR-15a-5p, and miR-183-5p (Figure 4B(i) and Table 5). In differentiating NSTEMI from stable angina patients, miR-183-5p scored the highest discriminatory power compared to the other miRNAs, with the highest AUC value of 0.917 (*p* < 0.0001; 95% CI, 0.680 to 0.995), a sensitivity value of 100% and a specificity of 83% (Figure 4B(ii) and Table 5).

### 2.5. Discriminatory Power of the miRNA Combination Panel

**Figure 5:**
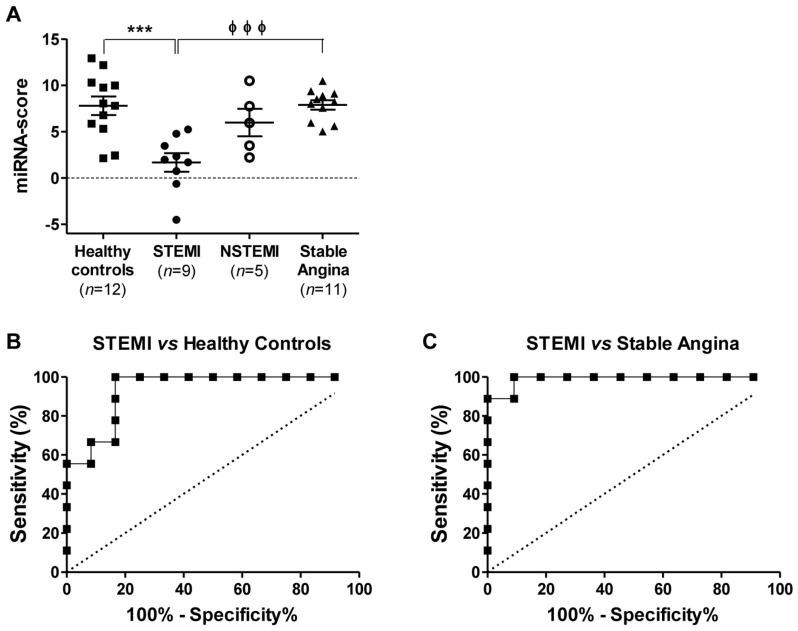
Discriminatory power of miRNAs combination panel for STEMI from stable angina patients. (**A**) miRNA-score of the miRNAs combination panel (miR-134-5p, miR-15a-5p, and let-7i-5p) is presented as means ± standard error of mean (SEM) in healthy controls (*n* = 12), patients with STEMI (*n* = 9), NSTEMI (*n* = 5), and stable angina (*n* = 11); *** indicates *p* < 0.001 compared to healthy control individuals; and ϕϕϕ indicates *p* < 0.001 compared to stable angina patients. Receiver operator characteristic (ROC) curves for the miRNAs combination panel in STEMI patients against (**B**) healthy controls or (**C**) patients with stable angina.

An miRNA combination panel consisting of miR-134-5p, mR-15a-5p, and let-7i-5p was evaluated for its ability in differentiating STEMI patients from the healthy controls (Figure 5). Prior to the analysis, an miRNA score of the miRNA combination panel was calculated as described in the Methods section. The combination had a higher discriminatory power in differentiating STEMI patients from the healthy controls, with an AUC value of 0.935 (*p* < 0.0001; 95% CI, 0.737 to 0.996), a sensitivity of 100% and a specificity of 83% (Figure 5B) when compared to that of the individual miRNAs (Figure 4A). Furthermore, this miRNA combination panel also improved the differentiation of STEMI patients from subjects with stable angina, with an AUC value of 0.990 (*p* < 0.0001; 95% CI, 0.814 to 1.000), a sensitivity of 100% and a specificity of 91% compared to that of the individual miRNAs (Figure 5C).

### 2.6. Correlation between Circulating miRNAs, Age, and Troponin I and CK-MB Levels

**Figure 6:**
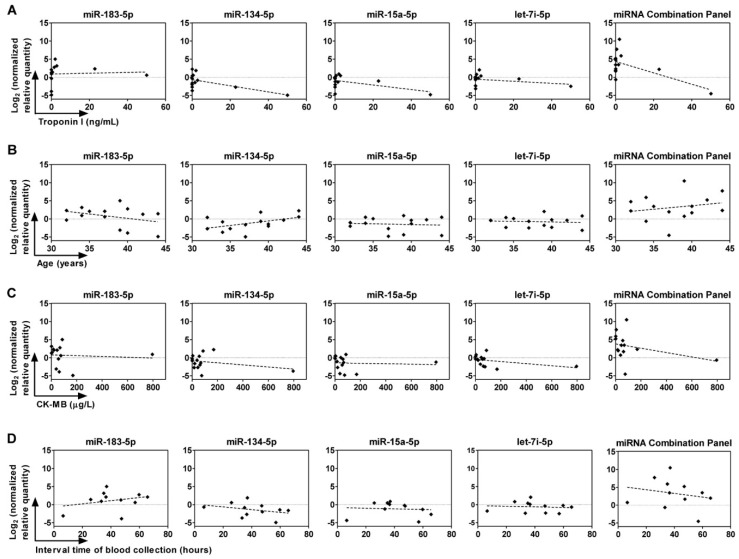
Correlation of plasma miRNAs with plasma cardiac biomarkers, age, and time of blood withdrawal. Pearson correlation analyses were conducted to determine the association of expressions of miRNAs with (**A**) troponin I levels, (**B**) age, (**C**) creatine kinase-myocardial band (CK-MB) levels in STEMI and NSTEMI patients, and (**D**) interval time of blood collection. The miRNA combination panel consists of miR-134-5p, mR-15a-5p, and let-7i-5p.

To determine whether the expression levels of miRNAs correlate with age, plasma troponin I, or CK-MB levels of STEMI and NSTEMI patients, Pearson correlation analyses were performed. The expression of miR-134-5p was inversely correlated with plasma troponin I levels in ACS patients (Pearson *r* = −0.586, *p* = 0.035, Figure 6A). In addition, correlation with plasma troponin I levels improved with the miRNA combination panel of miR-134-5p, miR-15a-5p, and let-7i-5p (Pearson *r* = −0.611, *p* = 0.027, Figure 6A). The expression of miR-134-5p was moderately, but not significantly (*p* = 0.07), correlated with the age of patients with a Pearson r value of 0.5 (Figure 6B). For plasma CK-MB levels or interval time of blood collection in ACS patients, no significant correlation was found with circulating amounts of miR-183-5p, miR-134-5p, miR-15a-5p, and let-7i-5p (Figure 6C,D).

### 2.7. Expressions of miRNAs in Tissues and Organs

**Figure 7:**
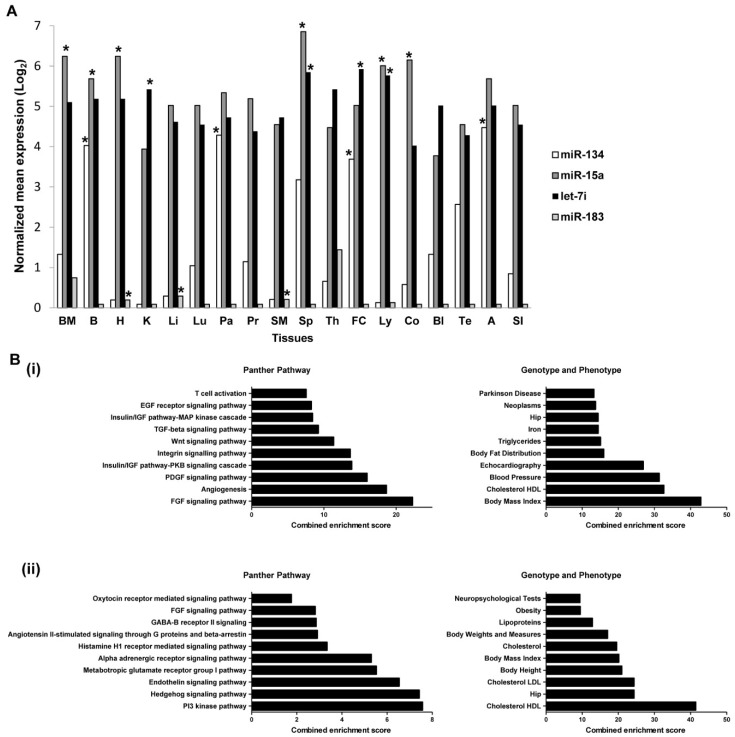
Expressions of miRNAs in tissues and enrichment analysis for predicted target genes of miRNA candidates. (**A**) Tissues expression analysis of miRNAs was performed using the online database of microRNA Expression and Sequence Analysis database (mESAdb) (http://konulab.fen.bilkent.edu.tr/mirna/mirna.php). * *p* < 0.05 depicts significant enrichment in the respective tissue. (**B**) Panther pathway and Genotypes and Phenotypes enrichment analysis for: (i) union set of predicted target genes for miR-134-5p, miR-15a-5p and let-7i-5p and (ii) predicted target genes for miR-183-5p were performed using the Enrichr online algorithm program. Combined enrichment scores which integrate *p*-value and *z*-score information as described in Section 4.8 were used to rank the enrichment term in analysis. The top 10 enrichment terms are reported for each bar graph. Abbreviations: BM, Bone Marrow; B, Brain; H, Heart; K, Kidney; Li, Liver; Lu, Lungs; Pa, Pancreas; Pr, Prostate; SM, Skeletal muscle; Sp, Spleen; Th, Thymus; FC, Frontal Cortex; Ly, Lymph node; Co, Colon; Bl, Bladder; Te, Testicle; A, Adrenal gland; SI, Small intestine; HDL, high-density lipoproteins; and LDL, low-density lipoproteins.

The expressions of miR-183-5p, miR-134-5p, miR-15a-5p, and let-7i-5p across different tissues were estimated, using the miRNA search analysis module on microRNA Expression and Sequence Analysis database (mESAdb) (http://konulab.fen.bilkent.edu.tr/mirna/mirna.php) to determine their potential tissues of origin [34]. The expression of miR-183-5p is the lowest across all tissues in comparison to miR-134-5p, miR-15a-5p, and let-7i-5p (Figure 7A). MiR-183-5p is significantly enriched in the heart, liver, and skeletal muscle (*p* < 0.05); miR-15a-5p is also significantly enriched in the heart as well as in the bladder, colon, kidney and the immune system tissues (bone marrow, lymph node, and spleen, *p* < 0.05) whereas both miR-134-5p and let-7i-5p are abundantly found in the brain and frontal cortex.

### 2.8. Predicted Target Genes of miRNAs Are Enriched in Metabolic Pathways

Significant deregulation of miR-134-5p, miR-15a-5p, and let-7i-5p was observed in young STEMI patients, therefore the union set of predicted genes for miR-134-5p, miR-15a-5p, and let-7i-5p were selected for enrichment analysis using Enrichr [35,36], whereby the predicted genes for miR-183-5p were submitted for enrichment analysis as described. Pathway enrichment analysis suggested that the predicted target genes of miR-134-5p, miR-15a-5p, and let-7i-5p were enriched in the Ras/MAPK, PI3K-AKT signaling pathway, angiogenesis, integrin, and Wnt signaling pathways which regulate cell proliferation, cell survival, and other basic developmental processes (Figure 7B(i)). Enrichment analysis in the Disease/Drug enrichment category in Enrichr suggested the presence of interaction between these predicted genes with most of the risk factors of cardiovascular disease such as low-density lipoprotein (LDL), blood pressure, and body fat distribution as listed in Genotypes and Phenotypes database (Figure 7B(i)). In addition, the predicted target genes of miR-183-5p include those in Hedgehog and endothelin signaling pathways, metabolic pathways, and are associated closely with HDL, LDL, and cholesterol metabolism (Figure 7B(ii)). The enrichment analysis of the list of predicted genes for miR-134-5p, miR-15a-5p, let-7i-5p, and miR-183-5p, hence revealed that these miRNAs and their predicted mRNA targets have roles aligned to events in the pathogenesis of ACS.

## 3. Discussion

The role of miRNAs in the pathophysiology of atherosclerosis and CAD has received increasing attention. MiRNAs modulate endothelial function, communication between endothelial cells and vascular smooth muscle cells, differentiation of the latter, and regulation of inflammatory cells in blood vessels, all of which are events leading to the development of CAD [37,38]. Deregulated expressions of cardiac specific miRNAs such as miR-1, miR-499, miR-133a/b, miR-208a/b, and miR-145 have been established in patients with stable CAD, ACS, and AMI, and their roles in events (regulation of endothelial function, differentiation of vascular smooth muscle cells, and plaques stabilization) leading to the development of these diseases are documented [39]. However, some cardiac-specific miRNAs such as miR-1, miR-133b, and miR-499 may have less discriminative power for AMI and unstable angina as they can also be released from injured skeletal muscles and thus conditions other than acute myocardial necrosis may influence their circulating levels [40]. Despite this limitation, in a large cohort of ACS patients, the levels of miR-132, miR-140-3p, and miR-210 predicted mortality, further highlighting the value of using miRNAs for disease prognosis [41], and underscoring the need for further identification of miRNAs for disease stratification and more accurate diagnosis.

The present study was undertaken to identify plasma miRNAs in young individuals diagnosed with ACS in Malaysia. From an initial list of top 13 up-regulated and 16 down-regulated miRNAs identified by sRNA-seq with differential expression analysis of FDR < 10% from the STEMI group, subsequent validation in the qRT-PCR cohort revealed significantly lower expressions of miR-134-5p, miR-15a-5p, and let-7i-5p in patients with STEMI, and higher expression of miR-183-5p in NSTEMI patients, compared to healthy controls. However, cardiac-specific miRNAs (e.g., miR-1, miR-208a, miR-208b, miR-133a, miR-133b, and miR-499), smooth muscle-enriched miR-143 and miR145 (released from injured cardiomyocytes), inflammation-associated miR-155, vascular protective miR-126 and endothelial-enriched miR-17 and 92a [40,42,43,44,45,46,47,48,49,50,51] were absent in the list of significantly deregulated plasma miRNAs as identified by sRNA-seq, possibly because of low sequencing reads. Indeed, qRT-PCR quantification revealed that these miRNAs are present in relatively low abundance in the plasma of ACS subjects [22]. 

Previously, let-7i-5p and miR-15a-5p were reported to be deregulated in CAD [52,53,54]. Significant down-regulation of let-7i was observed in elderly patients with stable CAD in comparison to healthy controls [52,54]. However, in the present cohort of young patients, let-7i-5p did not discriminate stable angina from healthy controls but instead identified the STEMI from the healthy control and stable angina groups. Members of the let-7 family share the same seed sequence and are conserved [55]. It has been reported that let-7g preserves endothelial function and suppresses inflammation induced by metabolic deregulation [56]. Indeed, pathway enrichment analysis showed that targets of let-7i-5p are involved in angiogenesis and metabolic pathways. In addition, this miRNA is also significantly enriched in lymph nodes and the spleen, which is suggestive of its potential role in inflammation.

The expression of miR-15a-5p is up-regulated in the plasma of elderly stable CAD (non-occlusive) patients when compared to healthy controls [53]. However, its differential expression was significantly different between the present young patients with stable angina (occlusive) and healthy controls. MiR-15a-5p has instead discriminated the STEMI from the healthy control and stable angina groups. These observations suggest that age factors or the differences between CAD presentations (occlusive versus non-occlusive) may lead to the differential expression of these miRNAs. Likewise, miR-15a-5p is also abundantly expressed in the bone marrow, lymph nodes and the spleen and, together with let-7i-5p and miR-134-5p, addresses predicted targets genes that are involved in endothelin signaling pathways, metabolic pathways, and lipid metabolism. In addition, miR-15a-5p is also significantly enriched in the heart, suggesting that it could potentially be cardio-specific. The ability of both let-7i-5p and miR-15a-5p in discriminating STEMI from stable angina and healthy controls warrants further evaluation of their potentials for the diagnosis of STEMI.

MiR-134-5p has been identified in plasma or serum of patients with STEMI, NSTEMI, unstable angina pectoris, acute myocardial infarction, and acute pulmonary embolism [17,18,20]. An earlier study showed a 6-fold increase in miR-134-5p expression, six hours after the onset of symptoms [57] while a kinetic study reported its peaked expression (6-fold) on admission [20]. In the present study, the expression of miR-134-5p was lower in STEMI and NSTEMI patients compared to healthy controls. The expression was measured approximately 36 h after the onset of symptoms, an average time needed for stabilization of the patients in the clinical ward, examination by ECG and percutaneous coronary angiogram. Nevertheless, the expression of miR-134-5p remained detectable and could discriminate the STEMI patients from healthy controls and stable angina patients with a relatively high AUC value, in agreement with a previous report [22]. Pearson analysis revealed no correlation between the expression of miR-134-5p and the time after the onset of symptoms at which blood was collected. However, one could still argue that a late sampling time may have contributed to the lower expression level of miR-134-5p in the present study, compared to earlier finding [22]. The kinetic study mentioned above [20] showed a 1.6-fold increase in expression 22 h after symptoms onset in patients with acute myocardial infarction; thus, despite a gradual decline in miR-134-5p expression with time, it still can be expected to be above baseline at the moment that blood was sampled for the present experiments. Hence, contrary to the higher expression reported in older ACS patient group [20,22], miR-134 levels are indeed lower in the young ACS group compared to healthy controls. MiR-134-5p accelerates atherosclerosis by promoting lipid accumulation and inflammatory responses following induction of lipoprotein lipase in macrophages [58], which explains its higher (3.5-fold) expression in peripheral blood mononuclear cells isolated from patients with unstable angina [17]. Endothelium-expressed miRNAs such as miR-126, -17, -92a, and inflammatory-associated miR-155 are also reduced in the blood of patients with stable CAD, suggesting that these miRNAs are taken up into the atherosclerotic lesions [52]. In this regard, it has been reported that macrophages in atherosclerotic lesions express scavenger receptors (SRs) for the uptake of oxidized low-density lipoproteins and several miRNAs have been shown to regulate SRs in atherosclerosis [59]. Hence, it appears worthwhile to investigate the role of miR-134-5p from this aspect as well. Combining miR-134-5p with miR-15a-5p and let-7i-5p improved the power of discriminating STEMI patients from those with stable angina with the highest AUC value compared to those of the individual miRNAs. This is not surprising given that each miRNA has multiple targets and multiple miRNAs can act in a cooperative way to modulate events related to CAD pathogenesis as indicated by the present target genes enrichment analysis. Thus, the use of a combination of multiple miRNAs could achieve greater diagnostic power for ACS.

MiR-183-5p plays an important role in maintaining endothelial barrier function and vascular permeability [60], even though it is better known as an oncomir [61,62,63,64] and is overexpressed in tumor tissues. MiR-183-5p regulates the tightness of the blood-nerve barrier [65] by regulating claudin-5 protein, an endothelial cell-specific tight junction component [66]. The expression of miR-183-3p is down-regulated in plasma of heart failure patients, suggesting that miR-183 could potentially be a marker of heart failure [67]. Indeed, the present tissue expression analysis revealed that miR-183 is enriched in the heart and skeletal muscle. The significant up-regulation of miR-183-5p in plasma of NSTEMI patients in the present study could potentially be related to its effects on the endothelial barrier, particularly on the tight junction proteins during atherogenesis, but this warrants future investigations. If indeed miR-183-5p is involved in atherogenesis, it could potentially serve as an early marker for ACS patients without malignancy. Nevertheless, since it is enriched in skeletal muscle, its circulating levels may be impacted by conditions involving injury of those tissues.

Several limitations of the present study merit consideration. First, the study was limited by a small cohort size given that we could recruit only a small number of patients below 45 years of age due to the relatively lower prevalence of ACS in younger age groups (23%) compared to older age groups (77%). Due to this low number, we were also unable to stratify the cohort according to ethnic groups to further identify ethnicity-related miRNAs. The expression of miR-375 and its CpG methylation is significantly different between the Han and Kazak ethnic groups of type 2 diabetes patients [68]. MiR-375 was also identified by the present sRNA-seq but further evaluation of its expression by qRT-PCR yielded insignificance. In another example, polymorphism within the miR-155 binding site on the 3′UTR of its target gene, angiotensin II type 1 receptor (*AGTR1*) has been identified in different ethnic groups. This polymorphism can disrupt miR-155 regulation of *AGTR1*, leading to increased expression which contributes to vasoconstriction and increases the risk for cardiovascular disease [69,70]. In Malaysia, the Indian ethnic group has the highest prevalence of ACS followed by the Malay and Chinese groups and hence, a larger cohort that enables ethnic group stratification would be required to determine whether or not the miRNAs identified in the present study are ethnically differently distributed.

Second, we observed discrepancies between the expression trend for miR-183-5p and miR-15a-5p analyzed by sRNA-seq and qRT-PCR. The sRNA-seq analysis showed up-regulation of miR-183-5p for STEMI patients but qRT-PCR validation revealed increased miR-183-5p expression only in NSTEMI patients. Likewise, for miR-15a-5p, the sRNA-seq analysis showed up-regulation in STEMI patients but the qRT-PCR validation instead revealed down-regulation in STEMI patients. These discrepancies probably reflect the higher sensitivity and sequence-specificity of qRT-PCR, which can quantify absolute copies numbers of specific mRNA or small RNA species, whereas, with the RNA-seq method, miRNA quantification is expressed as a value relative to the total number of sequences reads of a given sample. Hence, for miRNAs with a high variance in expression distribution across samples, comparisons between the latter may not yield reliable results [71]. 

Third, we did not perform statistical correction for confounding factors as the number of subjects in each group was low. It has been reported that plasma levels of cardiomyocyte-specific miRNAs such as miR-208b and miR-499 are not affected by a wide range of clinical confounders such as age, gender, body mass index, kidney function, systolic blood pressure, and white blood cell count [45]. However, confounding factors such as medication (including statins) influence the events involved in the pathophysiology of cardiovascular diseases by regulating the expression of particular miRNAs. For example, simvastatin can decrease miR-155 expression by disrupting the mevalonate-geranylgeranyl-pyrophosphate-RhoA signaling pathway leading to endothelial nitric oxide synthase expression and endothelium-dependent vasodilatation [72]. Atorvastatin induces let-7i expression and down-regulates toll-like receptor 4 signaling in monocytes from CAD patients thereby blunting immune responses [54]. Similarly, in statin-treated unstable angina patients, miRNAs such as miR-15b, -17, -20a and -93 that primarily target genes involved in angiogenesis are down-regulated, in line with the atheroprotective effects of statins in these patients [73]. Hence, these findings highlight the importance of statistical corrections for the effect of medications in future larger cohort studies. Last, the present study did not perform time course measurements of the changes in the levels of the identified miRNAs, which would provide the optimal detection window to identify their dynamic contribution.

## 4. Materials and Methods

### 4.1. Participants

Recruitment of participants was conducted at the University Malaya Medical Centre, Kuala Lumpur, Malaysia in accordance to the approved guidelines by the local Medical Ethics Committee (Ethics No. 889.6, November 2011). Informed consent was obtained from all subjects. 

For sRNA-seq, 14 ACS male patients below 45 years of age, clinically diagnosed with either NSTEMI or STEMI, and 14 gender- and age-matched control subjects were enrolled in this first cohort. The diagnosis of STEMI and NSTEMI in this study is based on local guidelines. STEMI is defined as persistent ST segment elevation ≥ 1 mm in two contiguous electrocardiographic leads, or the presence of a new left bundle branch block in the setting of positive cardiac markers. NSTEMI is defined as the occurrence of acute myocardial infarction in the setting of positive cardiac markers, with or without accompanying electrocardiographic changes other than ST-segment elevation [74,75]. The healthy controls had proven normal coronary via invasive percutaneous coronary angiogram.

For qRT-PCR validation, 14 ACS patients, 12 healthy control subjects and 12 aged-matched stable angina patients were recruited in this second cohort. The stable angina patients previously had CAD confirmed by percutaneous coronary angiogram but had not presented with acute episodes for more than one year. 

Exclusion criteria for the ACS and stable angina groups include previous history of cardiovascular diseases such as myocardial infarction, heart failure and cardiomyopathy. For the selection of healthy subjects, chronic alcoholics, smokers and those with family history of cardiovascular disease (CVD) or on CVD medications and familial hypercholesterolemia were excluded. Only males were included in the present study to preserve data homogeneity.

### 4.2. Isolation of Human Plasma

For confirmed cases of STEMI, NSTEMI and stable angina, blood collection was performed within 36 h after admission. Whole blood samples (6 mL) were collected by direct venous puncture into tubes containing ethylenediaminetetraacetic acid (EDTA), centrifuged at 2000× *g* for 15 min after which the supernatant (plasma) was carefully transferred into an RNase-free tube for extraction of miRNAs. The resultant plasma was aliquoted into cryopreservation tubes and stored at −80 °C until use. 

### 4.3. MiRNA Extraction

MiRNAs were extracted from plasma using the miRNeasy serum/plasma kit (Qiagen, Hilden, Germany), and finally eluted into 14 μL of RNase-free water according to the manufacturer’s instructions. Subsequently, the miRNA samples were concentrated using refrigerated CentriVap benchtop centrifugal concentrator (Labconco Corporation, Kansas City, MI, USA). An Agilent Small RNA Kit was used to verify miRNA quality and quantity using Agilent 2100 Bioanalyzer (Agilent Technologies, Santa Clara, CA, USA).

### 4.4. Small-RNA Sequencing (sRNA-seq)

Library preparation and sRNA sequencing was performed on each plasma sample using the Illumina platform, following the manufacturer’s protocol (Illumina Inc., San Diego, CA, USA). Small RNA libraries were constructed using a TruSeq Small RNA Sample Preparation kit (Illumina) according to the manufacturer’s instructions. Briefly, small RNA samples (18–30 nt) were gel-purified and ligated to the 5′ and 3′ adapters, followed by RT-PCR for cDNA library construction and incorporation of index tags. The cDNA library fragments were purified, separated on a PAGE gel and loaded on an Agilent Technologies 2100 Bioanalyzer to check size, purity, and concentration. Libraries were sequenced using Illumina HiSeq2000 at the BGI-Shenzhen (Shenzhen, Guangdong, China).

### 4.5. Sequencing Data Analysis

The read data were assessed and pre-processed using FastQC (http://www.bioinformatics.babraham.ac.uk/projects/fastqc) and FASTX-Toolkit (http://hannonlab.cshl.edu/fastx_toolkit), respectively. Briefly, the 3′ adapter sequences and low quality bases (<Q30) were trimmed. All reads that were shorter than 18 bp or containing unknown bases (N) were discarded. For each dataset, reads with identical sequences were collapsed to give a set of non-redundant reads (nr-reads). The nr-reads were then aligned to the human genome (GRCh37) using the mapper module from miRDeep2 [76] by allowing one mismatch. Unmapped reads/nr-reads or reads/nr-reads mapped to more than five regions in the human genome were filtered. The distribution of mapped reads was analyzed by cross-checking with miRBase v20 (http://www.mirbase.org/), Ensembl annotations and Rfam v11 database (http://rfam.xfam.org/). Finally, the miRDeep2 program (https://www.mdc-berlin.de/content/mirdeep2-documentation) [76] was used to identify known miRNAs (based on miRNA data from miRBase v20) to predict novel miRNAs and to summarize the read counts for each miRNA in every sample. The comma separated values (csv) files for miRNA expression from mirDeep2 were obtained for further analysis. Differential expression of the miRNA-Seq raw count data was assessed using the BioC/R package DESeq2 (https://bioconductor.org/packages/release/bioc/html/DESeq2.html) [32]. The data were adjusted to a common scale by normalizing them for different library sizes. The data (miRNA) dispersion from the mean was estimated, which provides the basis for inference. *p*-values were adjusted using multiple testing with the Benjamini and Hochberg (1995) approach by applying the FDR of 10% [77]. The list or miRNAs with adjusted *p*-values were filtered at *p* = 0.1. 

### 4.6. Validation of miRNA by qRT-PCR

To validate the result of sRNA-seq data analysis, two-step qRT-PCR was performed to quantitate the expression of selected miRNAs. For reverse transcription, a total input of 10 ng of total RNA was used to synthesize cDNA using a Taqman miRNA Reverse Transcription Kit according to the manufacturer’s instructions (Applied Biosystems, Foster City, CA, USA). Taqman miRNA Assay for miR-183-5p (Assay ID: 002269), miR-134-5p (Assay ID: 001186), miR-15a-5p (Assay ID: 000389), let-7i-5p (Assay ID: 002221), miR-375 (Assay ID: 000564) and miR-1307-5p (Assay ID: 47256_mat) were purchased from Applied Biosystems. Taqman Fast Advance PCR Master Mix (Applied Biosystems) was used to perform the real-time RT-PCR. As it is reported that specific small nuclear RNA and small nucleolar RNA such as RNU44, RNU68 and RNU6 are highly variable or not detectable in human plasma [61,62] and there is still no consensus on a suitable reference miRNA that can be used for qRT-PCR normalization, candidate reference miRNAs such as miR-320a (Assay ID: 002277) and miR-17-5p (Assay-ID: 000393) were selected as endogenous references based on their consistent expressions in sRNA-seq analysis. The expression stability of both reference miRNAs were also analyzed using geNorm and normalization by multiple reference genes was performed as proposed by Hellemans et al. (2007) [78,79]. Briefly, normalized relative quantity (NRQ) values were obtained by dividing the relative quantity (RQ) of the target miRNA with a normalizing factor (NF). NF was calculated from the geometric mean of RQ of the two reference miRNAs in each sample. RQ of target miRNAs in each sample was calculated using the equation: RQ = E^ΔCt^ and ΔCt = Ct_mean sample_ − Ct_sample_ whereby, E refers to the amplification efficiency of the primer and Ct refers to the cycle threshold value in qRT-PCR. Log_2_-transformed NRQ values, representing the expression of target miRNAs were used to plot graphs. 

### 4.7. Tissue Expression Analysis of miRNA Candidates

The expressions of miR-183-5p, miR-134-5p, miR-15a-5p, and let-7i-5p across different tissues were analyzed using the miRNA search analysis module on mESAdb [34] which is accessible at http://konulab.fen.bilkent.edu.tr/mirna/mirna.php. Normalized mean expressions obtained from a dataset comprising of normal human tissues and organs by Baskerville et al. [80] was analyzed. *p* < 0.05 depicts significant enrichment in the respective tissue. 

### 4.8. Target Genes Prediction and Gene List Enrichment Analysis for miRNA Candidates

The target genes of miR-183-5p, miR-134-5p, miR-15a-5p and let-7i-5p were predicted using TargetScan [81] database. The target genes predicted were selected for subsequent pathway and gene ontology enrichment analysis using Enrichr [35,36]. Adjusted *p*-values, *z*-score, and combined enrichment score were computed by Enrichr. The combined enrichment score was described as a combination of the *p*-value and *z*-score calculated as c = ln(p) × *z*, whereby, c is the combined enrichment score, *p* is the *p*-value computed by Fisher’s exact test and *z* is the *z*-score computed in order to access the deviation from the expected rank. The combined enrichment score was used to rank the enrichment terms in the analysis.

### 4.9. Statistical Analysis

Quantitative data were first evaluated to determine whether or not they followed Gaussian distribution using the D’Agostino and Pearson omnibus and Shapiro-Wilk normality test [82,83,84,85]. For *p*-value greater than the alpha level of 0.05, the null hypothesis that the data reflect a normally distributed population cannot be rejected. Therefore, one-way ANOVA followed by the Tukey multiple groups comparison test was performed for normally distributed data whereas the non-parametric Kruskal Wallis test was used for data not distributed normally. In addition, qualitative data were compared using the Chi-Square test. Statistical analysis and *p*-values were computed using GraphPad Prism version 5.0 (GraphPad Software, San Diego, CA, USA). Statistical significance of differences was accepted at *p* < 0.05. 

ROC curve analysis and AUC were performed to discriminate healthy controls from patients with STEMI, NSTEMI or stable angina and to investigate the diagnostic accuracy of selected miRNAs. Standard errors of AUCs and 95% confidence intervals (95% CI) were computed following the method of DeLong et al. (1988) [33]. Based on ROC analysis, the sensitivity and specificity of miRNAs were evaluated via cut-off points obtained from the Youden index computed using MedCalc software version 16.8.4 (MedCalc Software, Ostend, Belgium) [20,86]. In addition, correlation analyses between the miRNAs with patients’ age, plasma troponin I and CK-MB levels were evaluated using Pearson’s correlation coefficient. To perform ROC and Pearson’s correlation analyses for the miRNA combination panel (miR-134-5p, miR-15a-5p and let-7i-5p), a miRNA score representing the cumulative expression of each miRNA in the panel was computed first as described by Goren et al. (2012), whereby the score for each sample is the sum of the inverted-normalized signals of each individual miRNA adjusted by subtracting a constant (the minimal score) so that the range of scores starts at 0 [87]. 

## 5. Conclusions

In conclusion, findings from sRNA-seq and qRT-PCR revealed a novel miRNA expression profile in young individuals with ACS compared to those of previous studies in patients of older age groups. MiR-183-5p emerged as the circulating miRNA that can significantly discriminate NSTEMI from STEMI, stable angina patients or healthy normal individuals whereas miR-134-5p could discriminate STEMI patients from healthy individuals. In addition, the combination panel, which include miR-134-5p, miR-15a-5p, and let-7i-5p scored better discriminatory power in differentiating STEMI patients from healthy controls. Procedural intervention for STEMI and NSTEMI are different. Hence, miRNAs that can discriminate between the two will aid in obtaining accurate intervention decision. Nevertheless, before these miRNAs can be used in clinical settings for diagnosis and/or therapeutic decision-making, evaluation in future trials with greater statistical power is warranted. CAD-associated miRNAs could be used to complement rather than to replace current diagnostic procedures to achieve rapid and greater accuracy in the stratification of ACS. In addition, elucidation is needed of the roles of these miRNAs in endothelial dysfunction, inflammation, cholesterol metabolism, and atherogenesis to provide clearer understandings to their contribution to the development and progression of ACS. 

## Figures and Tables

**Table 1:** Clinical characteristics of acute coronary syndrome (ACS) patients and control subjects used for small-RNA sequencing.

	STEMI Patients (*n* = 11)	NSTEMI Patients (*n* = 3)	Healthy Controls (*n* = 14)	*p*-Value
Age (years)	36.36 ± 1.73	39.67 ± 2.96	35.55 ± 1.92	0.5851
Male sex (% male)	11 (100)	3 (100)	14 (100)	
Health history, *n* (%):				
Alcohol drinker	1 (9.09)	0 (0)	0 (0)	
Smokers	11 (100)	1 (33.33)	0 (0)	
Morbid Obesity	0 (0)	0 (0)	0 (0)	
Familial Hypercholesterolemia	1 (9.09)	0 (0)	0 (0)	
Hypertension	4 (36.36)	0 (0)	1 (7.14)	
Diabetes mellitus	1 (9.09)	1 (33.33)	1 (7.14)	
Family History of CVD	7 (63.64)	2 (66.67)	0 (0)	
^#^ Medications, *n* (%):				
Acetylsalicylic acid	9 (81.82)	2 (66.67)	0 (0)	
Lipid lowering drugs	10 (90.91)	2 (66.67)	0 (0)	
Beta-blocker	6 (54.55)	1 (33.33)	0 (0)	
Others	2 (18.18)	1 (33.33)	0 (0)	
Laboratory tests				
WBC (×10^9^/L)	12.63 ± 1.14	11.90 ± 1.79	8.68 ± 0.56	0.0110 *
Glucose (mmol/L)	6.19 ± 0.55	8.10 ± 2.61	6.26 ± 0.58	0.4087
TC (mmol/L)	4.83 ± 0.40	5.07 ± 0.45	5.26 ± 0.44	0.7581
HDL (mmol/L)	0.96 ± 0.07	0.78 ± 0.10	1.19 ± 0.09	0.0329 *
LDL (mmol/L)	3.14 ± 0.37	3.39 ± 0.30	3.33 ± 0.38	0.5610
TG (mmol/L)	1.61 ± 0.22	1.97 ± 0.52	1.65 ± 0.20	0.7493
Troponin I (ng/mL)	11.78 ± 4.89	1.94 ± 0.69	ND	ND
Creatine kinase (U/L)	1859.67 ± 619.44	496.00 ± 337.93	641.3 ± 495.96	0.1037
CK-MB (µg/L)	88.33 ± 25.69	28.00 ± 25.03	4.03 ± 2.07	0.0698

Abbreviations: CVD, cardiovascular disease; WBC, white blood cells; TC, total cholesterol; HDL, high-density lipoproteins; LDL, low-density lipoproteins; CK-MB, creatine kinase-myocardial band; ND, not determined. ^#^ Medications were given on admission/in the ward; * significantly different compared to healthy control.

**Table 2:** Clinical characteristics of ACS patients and control subjects used for quantitative reverse transcription-polymerase chain reaction (qRT-PCR) validation.

	STEMI Patients (*n* = 9)	NSTEMI Patients (*n* = 5)	Healthy Controls (*n* = 12)	*p*-Value
Age (years)	37.78 ± 1.29	37.80 ± 2.15	36.80 ± 1.83	0.8912
Male sex (% male)	9 (100)	5 (100)	12 (100)	
Health history, *n* (%):				
Alcohol drinker	1 (11.11)	0 (0)	0 (0)	
Smokers	8 (88.89)	2 (40.00)	0 (0)	
Morbid Obesity	0 (0)	0 (0)	0 (0)	
Familial Hypercholesterolemia	0 (0)	0 (0)	0 (0)	
Hypertension	0 (0)	0 (0)	1 (8.33)	
Diabetes mellitus	0 (0)	2 (40.00)	1 (8.33)	
Family History of CVD	5 (55.56)	3 (60.00)	0 (0)	
^#^ Medications, *n* (%):				
Acetylsalicylic acid	8 (88.89)	3 (60.00)	0 (0)	
Lipid lowering drugs	8 (88.89)	3 (60.00)	0 (0)	
Beta-blocker	7 (77.78)	2 (40.00)	0 (0)	
Others	1 (11.11)	1 (20.00)	0 (0)	
Laboratory tests				
WBC (×10^9^/L)	13.22 ± 1.63	12.02 ± 1.17	9.13 ± 0.74	0.0587
Glucose (mmol/L)	6.33 ± 0.49	9.04 ± 2.00	7.10 ± 0.71	0.1979
TC (mmol/L)	5.66 ± 0.62	5.46 ± 0.41	5.62 ± 0.49	0.9685
HDL (mmol/L)	1.14 ± 0.26	0.99 ± 0.12	1.27 ± 0.09	0.0841
LDL (mmol/L)	3.55 ± 0.68	3.80 ± 0.37	3.58 ± 0.46	0.7561
TG (mmol/L)	2.04 ± 0.29	1.46 ± 0.16	1.72 ± 0.27	0.9510
Troponin I (ng/mL)	6.29 ± 6.24	5.97 ± 4.23	ND	ND
Creatine kinase (U/L)	1526.89 ± 461.82	760.20 ± 452.37	910.50 ± 721.50	0.3601
CK-MB (µg/L)	140.28 ± 83.35	35.20 ± 17.47	5.55 ± 2.45	0.2262

Abbreviations: CVD, cardiovascular disease; WBC, white blood cells; TC, total cholesterol; HDL, high-density lipoproteins; LDL, low-density lipoproteins; CK-MB, creatine kinase-myocardial band; ND, not determined; ^#^ Medications were given on admission/in the ward.

**Table 3:** Significantly deregulated miRNAs in ST-segment elevated myocardial infarction (STEMI) patients compared to healthy controls.

miRNA	Base Mean	Log_2_ Fold Change	Fold Change	Regulation	*p*-Value	Adjusted *p*-Value
hsa-miR-183-5p_MIMAT0000261	133.430	2.232	4.699	Up	0.0000856	0.00317
hsa-miR-19a-5p_MIMAT0004490	269.760	1.732	3.322	Up	0.00379	0.03740
hsa-miR-15a-5p_MIMAT0000068	466.668	1.680	3.205	Up	0.00252	0.02723
hsa-miR-101-5p_MIMAT0004513	1857.448	1.053	2.075	Up	0.00942	0.07019
hsa-miR-101-3p_MIMAT0000099	1857.448	1.053	2.075	Up	0.00942	0.07019
hsa-miR-103a-3p_MIMAT0000101	5231.824	1.047	2.066	Up	0.00250	0.02723
hsa-miR-107_MIMAT0000104	5231.824	1.047	2.066	Up	0.00250	0.02723
hsa-miR-103a-2-5p_MIMAT0009196	5231.824	1.047	2.066	Up	0.00250	0.02723
hsa-miR-16-5p_MIMAT0000069	5923.048	1.037	2.052	Up	0.01173	0.07091
hsa-miR-16-5p_MIMAT0000069	5925.776	1.036	2.051	Up	0.01180	0.07091
hsa-miR-25-5p_MIMAT0004498	18,099.042	0.918	1.890	Up	0.00473	0.04118
hsa-miR-30e-5p_MIMAT0000692	2296.889	0.897	1.862	Up	0.01612	0.08520
hsa-miR-30a-5p_MIMAT0000087	3027.454	0.849	1.801	Up	0.01892	0.09657
CM000680.1_15285_novel	3128.361	−2.349	5.093	Down	0.0000004213	0.00006
CM000668.1_36492_novel	1075.400	−2.202	4.602	Down	0.00122	0.02578
hsa-miR-1307-5p_MIMAT0022727	404.613	−2.191	4.565	Down	0.00003364	0.00166
hsa-miR-375_MIMAT0000728	1029.877	−2.058	4.164	Down	0.00036	0.01060
hsa-let-7i-5p_MIMAT0000415	58,413.753	−1.910	3.759	Down	0.000009499	0.00070
hsa-miR-134-5p_MIMAT0000447	156.437	−1.802	3.486	Down	0.00258	0.02723
hsa-miR-328-5p_MIMAT0026486	116.074	−1.657	3.154	Down	0.01198	0.07091
hsa-let-7f-5p_MIMAT0000067	58,027.774	−1.445	2.723	Down	0.00046	0.01131
hsa-miR-320a_MIMAT0000510	4133.844	−1.432	2.698	Down	0.00185	0.02723
CM000668.1_35903_novel	361.953	−1.362	2.570	Down	0.01030	0.07084
hsa-miR-181b-5p_MIMAT0000257	1130.724	−1.259	2.393	Down	0.00413	0.03818
hsa-miR-181b-5p_MIMAT0000257	1155.505	−1.232	2.349	Down	0.00159	0.02723
hsa-miR-409-5p_MIMAT0001638	3315.486	−1.158	2.232	Down	0.00948	0.07019
hsa-miR-744-5p_MIMAT0004945	1996.177	−0.973	1.963	Down	0.01053	0.07084
hsa-miR-181a-5p_MIMAT0000256	34,050.461	−0.656	1.575	Down	0.01379	0.07560
hsa-miR-181a-5p_MIMAT0000256	34,050.461	−0.656	1.575	Down	0.01379	0.07560

**Table 4:** Discriminatory powers of plasma miRNAs for STEMI and NSTEMI patients against healthy controls.

Patients	miRNAs	AUC	95% CI	*p*-Value	Sensitivity (%)	Specificity (%)
STEMI	miR-183-5p	0.509	0.285 to 0.731	0.947	33.3	83.3
	miR-134-5p	0.796	0.566 to 0.938	0.004 **	66.7	83.3
	miR-15a-5p	0.796	0.566 to 0.938	0.003 **	100.0	58.3
	let-7i-5p	0.833	0.608 to 0.958	0.0005 **	100.0	66.7
	miR-375	0.620	0.386 to 0.820	0.335	88.9	41.7
	miR-1307-5p	0.698	0.455 to 0.880	0.103	100.0	58.3
NSTEMI	miR-183-5p	0.917	0.680 to 0.995	<0.0001 ***	100.0	75.0
	miR-134-5p	0.717	0.451 to 0.904	0.144	60.0	83.3
	miR-15a-5p	0.617	0.355 to 0.836	0.411	100.0	50.0
	let-7i-5p	0.583	0.325 to 0.812	0.592	80.0	83.7
	miR-375	0.683	0.418 to 0.882	0.207	60.0	83.3
	miR-1307-5p	0.667	0.402 to 0.871	0.205	100.0	50.0

Abbreviations: AUC, area under the curve of ROC; 95% CI, confidence interval. *P*-values and 95% confidence intervals (95% CI) were computed following the method of DeLong et al. (1988) [33]. ** indicates *p* < 0.01; ***, *p* < 0.001.

**Table 5:** Discriminatory powers of circulating miRNAs for STEMI and NSTEMI patients against stable angina patients.

Patients	miRNAs	AUC	95% CI	*p*-Value	Sensitivity (%)	Specificity (%)
STEMI	miR-183-5p	0.546	0.318 to 0.761	0.756	55.6	75.0
	miR-134-5p	0.769	0.535 to 0.922	0.027 *	55.6	100.0
	miR-15a-5p	0.907	0.700 to 0.989	<0.0001 ***	100.0	66.7
	let-7i-5p	0.929	0.722 to 0.995	<0.0001 ***	100.0	81.8
NSTEMI	miR-183-5p	0.917	0.680 to 0.995	<0.0001 ***	100.0	83.3
	miR-134-5p	0.633	0.370 to 0.848	0.486	40.0	100.0
	miR-15a-5p	0.717	0.451 to 0.904	0.100	100.0	50.0
	let-7i-5p	0.618	0.348 to 0.843	0.525	60.0	81.8

Abbreviations: AUC, area under the curve of ROC; 95% CI, confidence level. *P*-values and 95% CI were computed following the method of DeLong et al. (1988) [33]. * indicates *p* < 0.05; ***, *p* < 0.001.

## Abbreviations

CAD	Coronary artery disease
ACS	Acute coronary syndrome
STEMI	ST-segment elevated myocardial infarction
NSTEMI	Non-ST-segment elevated myocardial infarction
ECG	Electrocardiography
CK-MB	Creatine kinase-myocardial band
RT-PCR	Reverse transcription-polymerase chain reaction
HDL	High-density lipoproteins
sRNA-seq	Small RNA sequencing
FDR	False discovery rate
ANOVA	Analysis of variance
ROC	Receiver operating characteristic
AUC	Area under the curve
95% CI	95% confidence interval
mESAdb	microRNA Expression and Sequence Analysis database
LDL	Low-density lipoproteins
VSMC	Vascular smooth muscle cells
SRs	Scavenger receptors
*AGTR1*	Angiotensin II type 1 receptor
CVD	Cardiovascular disease
EDTA	Ethylenediaminetetraacetic acid
NRQ	Normalized relative quantity
RQ	Relative quantity
NF	Normalizing factor
